# Childhood Trauma in Patients With PAH—Prevalence, Impact on QoL, and Mental Health—A Preliminary Report

**DOI:** 10.3389/fpsyt.2022.812862

**Published:** 2022-02-10

**Authors:** Da-Hee Park, Tanja Meltendorf, Kai G. Kahl, Jan C. Kamp, Manuel J. Richter, Henning Gall, Hossein A. Ghofrani, Marius M. Hoeper, Karen M. Olsson, Jan Fuge

**Affiliations:** ^1^Department of Respiratory Medicine, Hannover Medical School, Hannover, Germany; ^2^Biomedical Research in Endstage and Obstructive Lung Disease Hannover, German Center for Lung Research, Hannover, Germany; ^3^Department of Psychiatry, Social Psychiatry and Psychotherapy, Hannover Medical School, Hannover, Germany; ^4^Department of Internal Medicine, Justus Liebig University Giessen, Giessen, Germany; ^5^German Center for Lung Research, Universities of Giessen and Marburg Lung Center, Giessen, Germany

**Keywords:** pulmonary arterial hypertension, child maltreatment, mental health, quality of life, prevalence, pulmonary hypertension

## Abstract

**Background/Objective:**

Child maltreatment is associated with increased risk of psychological consequences, contributes to morbidity and has long lasting effects on mental health and quality of life. Child maltreatment has not been assessed in patients with pulmonary arterial hypertension (PAH). We examined the prevalence of child maltreatment and determined their impact on disease severity in patients with PAH.

**Methods:**

A cross-sectional observational multicenter study at two PH centers in Germany was conducted. Patients with a confirmed diagnosis of PAH were given a self-administered questionnaire. Child maltreatment using the Childhood Trauma Questionnaire (CTQ), quality of life (QoL), anxiety, depression, and lifestyle factors were assessed and enhanced by clinical parameters 6-min walk distance (6MWD), WHO functional class (WHO FC), and serum levels of N-terminal fragment of pro-brain natriuretic peptide (NT-proBNP). Prevalence rates of child maltreatment were compared to the general population and impact of child maltreatment on disease severity was calculated by logistic regression analysis.

**Results:**

Two-hundred and seventeen patients, 71% female and a median age of 56 years were enrolled in this study. Patients with PAH had higher rates of emotional abuse and lower rates of physical neglect compared to the German population while rates of emotional neglect, physical abuse, and sexual abuse did not differ between patients and German population. Patients with any form of child maltreatment were more likely to be active smokers, had a worse QoL and more anxiety or depression. Moderate associations between child maltreatment, mental health, QoL, lifestyle factors and clinical parameters could be observed. Logistic regression analysis showed a significant impact of CTQ-total score on disease severity with an OR of 1.022 (95%-CI: 1.001–1.042, *p* = 0.035).

**Conclusion:**

We found a higher rate of child maltreatment in patients with PAH in comparison to the German population. Correlations suggest moderate associations between CTQ-scores and mental health as well as QoL. Child maltreatment had significant impact on disease severity. However, effects were moderate. We conclude that child maltreatment has effects on mental health and quality of life in patients with PAH and may have limited effect on disease severity.

## Introduction

Pulmonary arterial hypertension (PAH) is progressive pulmonary vascular disease. Characterized by remodeling of pulmonary vasculature it leads to increased pulmonary vascular resistance (1, 2). Patients with PAH are experiencing symptoms such as dyspnea on exertion, fatigue and with disease progression potentially clinical signs of heart failure. These debilitating symptoms impair physical activities and quality of life (QoL) (3, 4). While the disease remains fatal as none of the currently available drugs are curative, improvement of therapeutic options, clinical outcomes and life expectancy have put questions of patient's quality of life, mental health, emotional, and social burden are on the forefront of current research (5–8). Data on prevalence of psychiatric disorders in PAH patients (9, 10) is still scarce. Olsson et al. demonstrated a higher prevalence of anxiety and depression disorders with negative impact on QoL in PAH compared to levels in the German population (11, 12). Child maltreatment may pose an additional risk factor associated with lower QoL and mental disorders in patients with PAH. Several studies have identified child maltreatment as an important contributing factor to the incidence and severity of depressive disorders (13). Child maltreatment is defined as the abuse and neglect against a child under the age of 18 by a parent or other caregivers. It includes all types of physical and/or emotional ill-treatment, sexual abuse and neglect which results in actual or potential harm to the child's health, survival, development, or dignity in the context of a relationship of responsibility, trust, or power (14). It has been associated with poorer overall health, greater physical, and emotional functional disability and higher degree of health risk behavior. Patients with multiple types of maltreatment showed greatest worsening of self-reported health symptoms (15). Child maltreatment is associated with various diseases such as the development of cardiovascular diseases, type 2 diabetes or chronic obstructive pulmonary diseases leading to higher mortality (16–18). Proskynitopoulos et al. (19) found increased prevalence of child maltreatment in adults with congenital heart disease compared to the general German population. Higher total Child trauma questionnaire (CTQ) scores correlated with decreased quality of life, higher rates of anxiety, and depression and contributed to the prediction of New York Heart Association (NYHA) scores (19). The present study aimed to assess the (i) prevalence of child maltreatment in patients with PAH and (ii) to compare this with the prevalence in the German general public as well as (iii) to assess the impact, differences and associations of child maltreatment on disease severity, lifestyle factors, mental health, and QoL in patients with PAH.

## Methods

This cross-sectional observational multicentre study included patients with a confirmed diagnosis of PAH at two participating PH referral centers (Hannover Medical School and University of Giessen and Marburg, both in Germany). The study concept was developed in cooperation with patient organizations as suggested during the latest Pulmonary Hypertension World Symposium in 2018 (20). All patients gave written informed consent. The study was approved by local institutional review boards (Nr. 8540_BO_K_2019 for Hannover and Nr. 21119 for Giessen and Marburg). Patients were contacted between September 2019 and March 2020. This study was conducted by a self-administrated questionnaire.

### Patient Setting and Clinical Parameters

Patients were selected based on diagnosis of PAH according to current criteria (1) and age ≥ 18 years. Assessment included hemodynamics from right heart catherization at time of diagnosis, 6-minute walk distance (6MWD), WHO functional class (FC), and serum levels of N-terminal fragment of pro-brain natriuretic peptide (NT-proBNP). Risk assessment for our cohort was based on three variables FC, 6MWD, and BNP/NT-proBNP as previously described (21, 22). Each variable was graded with a number as low risk (1), intermediate risk (2), and high risk (3). The average risk was calculated by dividing the sum of the grades by the number of available variables and rounding to the next integer (23).

### Assessment of Child Maltreatment

Child maltreatment was assessed using the German version of the Childhood Trauma Questionnaire CTQ (24). The CTQ consists of a 28-item self-rating scale divided in five subscales inquiring five areas: emotional abuse, physical abuse, sexual abuse, emotional neglect, and physical neglect. The items are rated from 1 (never true) to 5 (very often true). The subscale scores range from 5 to 25 with an overall score from 25 to 125 points. Several studies have supported the reliability and validity of reported childhood trauma obtained with this instrument, discriminant validity with structured trauma interviews and corroboration with independent data (24). Thresholds presented by Walker et al. (15) were used to construct groups with either form of maltreatment or no maltreatment. Child maltreatment was classified when threshold scores for emotional abuse (10), emotional neglect (15), physical abuse (8), physical neglect (8), and sexual abuse (8) were met. The CTQ-derived prevalence rates and mean-values of child maltreatment were compared to the German general population described by Iffland et al. (25).

### Assessment of Symptoms of Anxiety and Depression and Quality of Life

Symptoms of anxiety and depression were assessed with participants completing the Hospital Anxiety and Depression Scale (HADS) (26). The HADS questionnaire is divided into subscales for anxiety (HADS-A) and depression (HADS-D), each containing seven questions for a maximum of 21 points per subscale. The higher score indicating a more severe anxiety or depression. Zigmond et al. (26) proposed a score >11 per subscale to be associated with significant anxiety or depression. A cut-off score of 8 or above for both HADS-A and HADS-D was validated, showing probable signs of anxiety or depression (27, 28). The World Health Organization Quality of Life [WHO-QoL-BREF (29)] was used to assess quality of life.

### Statistical Analysis

IBM SPSS Statistics (version 28.0, IBM Corp., Armonk, New York) and Stata 13.0 (State Corp LP, College Station, Texas, USA) statistical software programs were used for statistical analysis. Continuous parameters are presented as median and interquartile range (Q_25_-Q_75_) or as mean and standard deviation (SD). Categorical variables are presented as number (*n*) and percent (%). Comparisons of continuous parameters were conducted using *t*-test or Mann-Whitney *U*-test (if not normally distributed or ordinal scaled) and comparison of categorical parameters by using Chi-square-test or fisher's exact test, unless indicated otherwise. Correlations of continuous and ordinal parameters were assessed using Pearson correlation coefficient. Parameters were CTQ-scores, lifestyle factors (exercise, drinks per week, and smoking intensity) and clinical parameters such as BMI, HADS-scores, QoL, 6MWD, and WHO FC. Logistic regression analysis was conducted to assess impact of CTQ-score on clinical outcome parameter WHO FC as surrogate for disease severity (WHO FC I and II vs. WHO FC III and IV was used as binary outcome). Further univariate logistic regression was conducted to assess impact of child maltreatment (any positive CTQ-subscore) on anxiety or depression disorder. Nagelkerke's R2 was determined to assess model quality. All tests were two-sided, *p* < 0.05 were considered statistically significant. *P*-values of multiple correlations were adjusted using the Benjamini–Hochberg-procedure.

## Results

**Figure 1:**
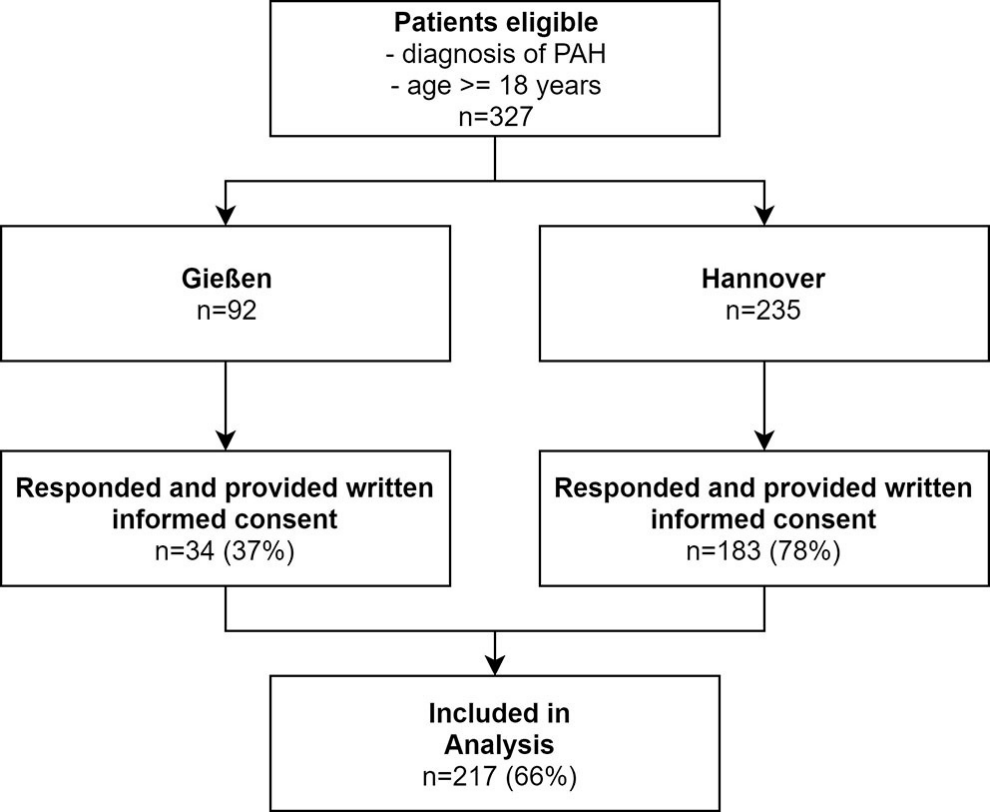
Flowchart of patient inclusion.

This patient cohort was previously described in the PEPPAH-study (12). A total of 327 patients were approached in this study. Two hundred and seventeen patients (66%) agreed to participate and returned the questionnaires (Figure 1). Patient characteristics are shown in Table 1. The majority of patients were female (*n* = 155, 71%); median age was 56 (49-67) years. All patients were treated with PAH medications, most of them (47%) with dual combination therapy. Nearly all patients (96%) fell in the low or intermediate ERS risk score category. Patients with any form of child maltreatment were more likely to be active smokers, had a worse overall QoL and more anxiety and depression. Trends could be observed in 6MWD with more than 30 m difference in patients with or without any form of child maltreatment (Table 1). Disease severity indicated by ERS risk status, WHO FC and NTproBNP, hemodynamics at time of diagnosis, and PAH treatment did not differ between groups.

**Table 1:** Patient characteristics.

	**All patients *n* = 217**	**Patients with any CTQ positive subscore *n* = 108 (50%)**	**Patients without any CTQ positive subscore *n* = 109 (50%)**	***p*-value of group comparison**
Age—years	56 (49-67)	55 (41-65)	57 (45-66)	0.422
Sex—female (%)	155 (71%)	74 (69%)	81 (74%)	0.345
BMI (kg/m^2^)	26 (23-31)	26 (23-31)	26 (23-31)	0.813
Time since PAH diagnosis (years)	6 (3-11)	6 (2-11)	7 (4-13)	0.062
**Diagnosis—*****n*** **(%)**				
-1.1 IPAH	122 (56%)	66 (61%)	56 (51%)	0.233^c^
-1.2 HPAH	25 (12%)	13 (12%)	12 (11%)	
-1.4 Associated PAH	70 (32%)	29 (27%)	41 (38%)	
**WHO FC**				
-I, *n* (%)	20 (9%)	7 (7%)	13 (12%)	0.439^c^
-II, *n* (%)	92 (43%)	45 (42%)	47 (44%)	
-III, *n* (%)	93 (43%)	51 (47%)	42 (39%)	
-IV, *n* (%)	10 (5%)	5 (5%)	5 (5%)	
**ESC/ERS risk status—*****n*** **(%)**				
-Low	107 (50%)	52 (49%)	55 (52%)	0.799^c^
-Intermediate	97 (46%)	51 (53%)	46 (47%)	
-High	9 (4%)	4 (44%)	5 (56%)	
6MWD	439 (353–521)	423 (339–502)	454 (371–539)	0.061
NT-proBNP (ng/l), *n* = 192	184 (88–517)	169 (74–419)	207 (104–586)	0.961
DLCO (% pred.), *n* = 138	62 (47-74)	64 (42-73)	61 (48-74)	0.844
paO_2_, mmHg	67 (60–75)	67 (60–76)	67 (61–75)	0.788
**Hemodynamics at diagnosis**				
-mPAP (mmHg)	48 (41-57)	48 (41-58)	49 (41-58)	0.651
-PAWP (mmHg)	9 (6-12)	9 (6-12)	9 (6-12)	0.794
-CI (l/min/m^2^)	2.4 (2.0–2.9)	2.4 (2.0–3.0)	2.4 (2.0–2.7)	0.284
-PVR (dyn·s·cm^−5^)	707 (501–947)	689 (505–951)	752 (498–927)	0.914
**PH-therapy—*****n*** **(%)**				
-Monotherapy	44 (20%)	21 (19%)	23 (21%)	0.673^c^
-Double combination therapy	102 (47%)	54 (50%)	48 (44%)	
-Triple combination therapy	71 (33%)	33 (31%)	38 (35%)	
**Smoking status**				
-Active, *n* (%)	24 (11%)	17 (16%)	7 (6%)	0.021^c^
-Former, *n* (%)	31 (14%)	19 (18%)	12 (11%)	
-Never, *n* (%)	162 (75%)	72 (67%)	90 (83%)	
-Packyears	14 (5-25)	15 (6-23)	9 (4-33)	0.495
Alcohol drinking (drinks per week)	0.8 ± 2.0	0.9 ± 2.5	0.8 ± 1.1	0.772^a^
Exercise score (points)	3 (2-4)	3 (2-3)	3 (2-4)	0.906^b^
HADS-A (points)	6 (2-9)	7 (3-10)	5 (2-9)	**0.034**
HADS-D (points)	5 (2-8)	6 (3-9)	4 (2-7)	**0.002**
QoL-overall (points)	50 (38-75)	50 (38-63)	63 (44-75)	**0.026**
QoL-psych (points)	71 (58–79)	63 (50-79)	71 (63–83)	**0.020**
QoL-physical (points)	57 (45-75)	57 (43-71)	64 (50-75)	**<0.001**
QoL-social (points)	67 (58-83)	67 (58-83)	67 (50-75)	**0.009**
QoL-environment (points)	78 (69–88)	75 (63–84)	78 (70–88)	**0.012**

*Continuous variables are stated as median and interquartile ranges (IQR) and categorical variables are stated as n and percent (%), unless indicated otherwise. All p-values were derived using t-test, unless indicated otherwise*.

a *Mean and SD because of distribution of the data*.

b *Non-parametric Mann–Whitney U-test because of ordinal scale of the variable*.

c *Chi^2^-test because of the categorical scale of the variable*.

*BMI, body mass index; QoL, quality of life; HADS-A, Hospital Anxiety and Depression Scale—anxiety; HADS-D, Hospital Anxiety and Depression Scale—Depression; I/HPAH, idiopathic or heritable pulmonary arterial hypertension; WHO FC, World Health Organization Functional Class; 6MWD, 6-min walking distance; NT-proBNP, N-terminal fragment of pro-brain natriuretic peptide; DLCO, diffusion capacity of the lung for carbon monoxide; paO_2_, mmHg, arterial pO_2_; mPAP, mean pulmonary arterial pressure; PAWP, pulmonary artery wedge pressure; CI, cardiac index; PVR, pulmonary vascular resistance; IQR, interquartile range. Statistically significant values as bold*.

### Prevalence of Child Maltreatment in Patients With PAH

**Figure 2:**
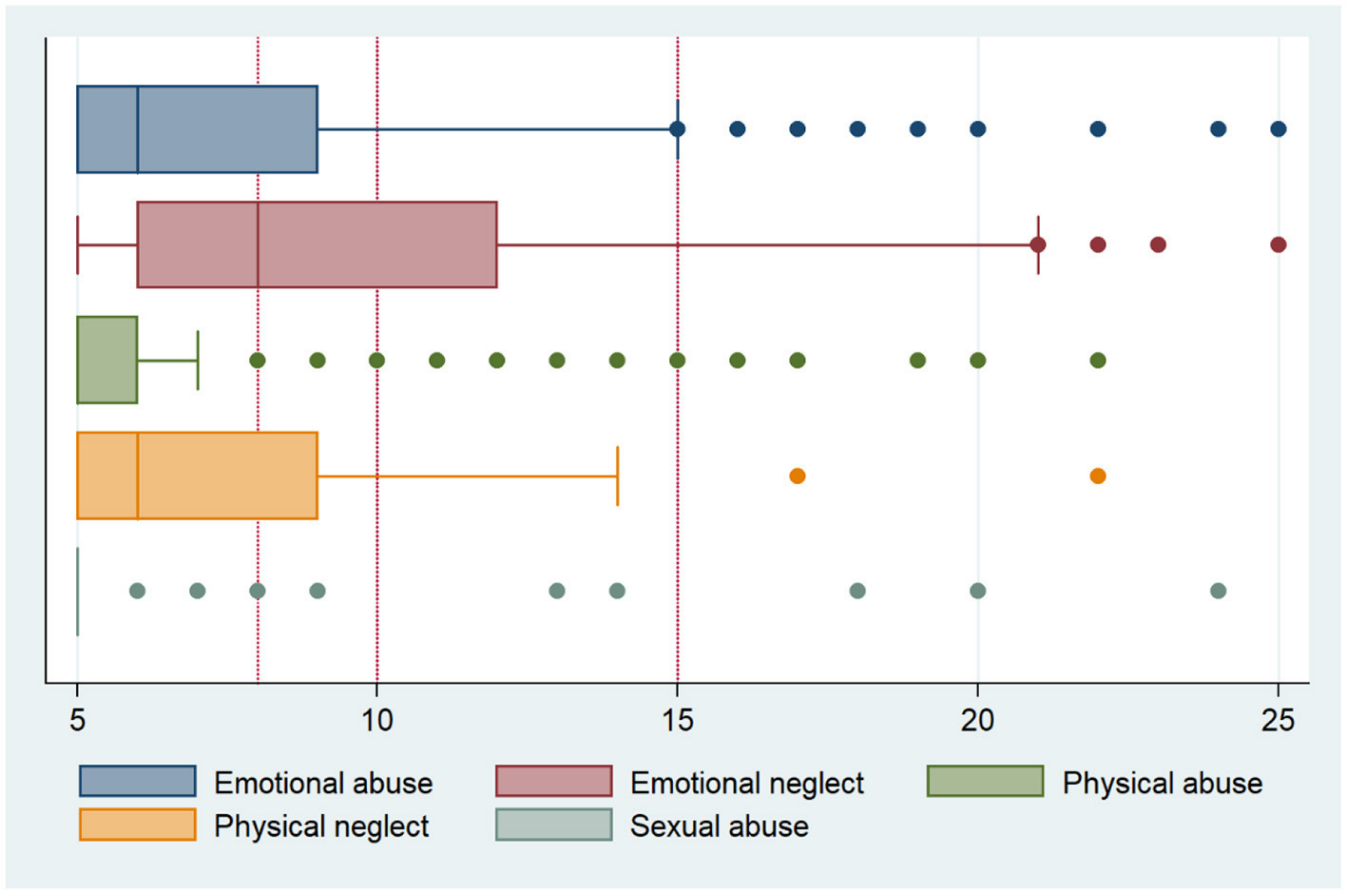
Subscores of childhood trauma questionnaire of patients with pulmonary arterial hypertension.

In this study, child maltreatment with a least one subgroup above threshold was detected in fifty percent of the patients (Table 1). Emotional abuse and emotional neglect were about 2 times more frequent in patients with PAH than in the general population (Table 1; Figure 2). Patients with PAH had a significantly higher total CTQ score (*p* < 0.001), while physical neglect was more prevalent in the general German population (48.4 vs. 36.4%, *p* = 0.041) and physical abuse did not significantly differ between both groups (14.3 vs. 12.0%, *p* = 0.458). Sexual abuse was reported under 10% in both patients with PAH and the general population (Table 2).

**Table 2:** Scores and prevalence of child maltreatment in patients with PAH compared to German population.

**Item**	**PAH**	**German population (25)**	***p***
	***n* = 217**	***n* = 2,500**	
**Comparison of CTQ-scores, Mean (SD)**
-CTQ-total score	41.84 ± 14.43	35.99 ± 10.48	**<0.001**
-CTQ-emotional abuse	7.68 ± 4.00	6.51 ± 2.60	**<0.001**
-CTQ-emotional neglect	9.58 ± 4.88	10.09 ± 4.23	0.093
-CTQ-physical abuse	6.19 ± 2.83	5.88 ± 2.17	**0.049**
-CTQ-physical neglect	7.18 ± 2.75	8.15 ± 3.02	**<0.001**
-CTQ-sexual abuse	5.54 ± 2.24	5.45 ± 1.66	0.458
**Comparison of CTQ-categories**, ***n*** **(%)**
-CTQ-emotional abuse	43 (19.8%)	254 (10.2%)	**<0.001**
-CTQ-emotional neglect	42 (19.4%)	348 (13.9%)	0.078
-CTQ-physical abuse	31 (14.3%)	301 (12.0%)	0.458
-CTQ-physical neglect	79 (36.4%)	1,210 (48.4%)	**0.041**
-CTQ-sexual abuse	14 (6.5%)	156 (6.2%)	0.920

*Continuous variables are stated as mean and standard deviation and categorical variables are stated as n and percent (%)*.

*CTQ, childhood trauma questionnaire; PAH, pulmonary arterial hypertension; SD, standard deviation; Statistically significant values as bold*.

### Correlation of CTQ Subscores on Quality of Life, Mental Health, Lifestyle, and Clinical Parameters

Moderate associations between CTQ-scores, mental health, QoL, lifestyle factors, and clinical parameters could be observed (see Table 3). Higher CTQ emotional-, physical, or total scores were correlated with higher HADS depression and anxiety scores (*r* = 0.201–0.401, *p* < 0.001) while being negatively correlated with QoL (*r* = −0.200 to −0.315, *p* < 0.001). History of smoking and WHO FC were positively correlated with emotional and physical CTQ-scores (*r* = 0.113–0.216). Total CTQ-scores were negatively correlated to 6MWD (*r* = −0.145 to *r* = −0.204, *p* < 0.005) whereas exercise and drinks per week were not significantly related. There were no significant association between CTQ-sexual abuse and any evaluatedparameter.

**Table 3:** Correlations of CTQ-scores in PAH with HADS, QoL, Lifestyle and 6MWD, and WHO FC.

	**HADS-D**	**HADS-A**	**QoL**	**BMI**	**Exercise**	**Drinks**	**Smoking**	**WHO FC**	**6MWD**
CTQ-total score	0.368^**^	0.351^**^	−0.315^**^	0.158^*^	−0.068	−0.071	0.188^*^	0.186^*^	−0.192^*^
CTQ-emotional abuse	0.388^**^	0.401^**^	−0.302^**^	0.126	−0.039	−0.027	0.181^**^	0.125	−0.174^*^
CTQ-emotional neglect	0.316^**^	0.255^**^	−0.308^**^	0.121	−0.056	−0.082	0.113	0.210^*^	−0.145^*^
CTQ-physical abuse	0.262^**^	0.268^**^	−0.200^*^	0.139^*^	−0.081	−0.066	0.157^*^	0.118^*^	−0.146^*^
CTQ-physical neglect	0.251^**^	0.204^*^	−0.269^*^	0.131	−0.041	−0.088	0.216^*^	0.192^*^	−0.204^*^
CTQ-sexual abuse	0.112	0.106	−0.056	0.123	−0.033	0.001	0.021	0.049	−0.097

*Pearson correlation coefficient. Statistically significant values are marked using asterisk*,

** *p < 0.001;*

* *p < 0.05*.

*BMI, body mass index; CTQ, childhood trauma questionnaire; QoL, quality of life; HADS-A, Hospital Anxiety and Depression Scale—anxiety; HADS-D, Hospital Anxiety and Depression Scale—Depression; PAH, pulmonary arterial hypertension; WHO FC, World Health Organization Functional Class; 6MWD, 6-min walking distance*.

### Impact of CTQ on Disease Severity and Mental Health

Univariate logistic regression analysis on disease severity (WHO FC I+II vs. WHO FC III+IV) revealed a significant impact of CTQ-total score with an OR of 1.022 (95%-CI: 1.001–1.042, *p* = 0.035) while the model describes 2.9% of the independent variable. Further univariate regression analysis showed that patients with reported emotional neglect had a 6.8% higher chance (95%-CI: 1.009–1.131, *p* = 0.023) of being in WHO FC III or IV per CTQ point increase. Model accuracy was 3.3%. Impact of any positive CTQ-subscore on anxiety had an OR of 1.678 (95%-CI: 0.788–3.574, *p* = 0.179) while impact on depression showed an OR of 2.761 (95%-CI: 1.369–5.209, *p* = 0.004) while model accuracy was 1.5 and 6%, respectively.

## Discussion

To the best of our knowledge this is the first study to assess the prevalence of child maltreatment in patients with PAH. Patients with PAH had a higher overall CTQ-score with fifty percent of our patients exceeding the threshold for any subscore of child maltreatment. In these patients, moderate associations between CTQ-scores and QoL, anxiety and depression, WHO FC, and 6MWD could be detected. Higher prevalence of CTQ-scores were mainly driven by higher subscores in emotional abuse or physical abuse. This is in line with previous observations from patients with congenital heart disease (19). While absolute scores were higher in patients with PAH, both groups reported only moderately elevated scores on the scale of 5–25 points per subscore (as seen in Figure 2). Inversely physical neglect was comparatively more frequent in the general population than in patients with PAH. It has been discussed that elderly who experienced privation of (post-)war year were more likely to report physical neglect (25). With comparatively weak psychometric properties and weak internal consistency, comparisons for the physical neglect subscale discussion on impact can only be held cautiously.

Several severe long-term consequences of child maltreatment have been studied and suggested. Ranging from mentioned physical health, psychological to behavioral, and societal consequences. Most children who experienced child maltreatment are often affected by many other adversities such as domestic violence, poverty, and parental substance abuse (30).

Child maltreatment poses a higher risk on the development of mental disorders such as depression (31–34). Olsson et al. have recently demonstrated a higher prevalence of mental disorders in patients with PAH. Our findings indicate a correlation between higher CTQ scores and higher HADS depression and anxiety scores. Gamble et al. (35) in as early as 2006 had described the correlation between childhood trauma and severity of anxiety and depression. Similarly, we demonstrated impact of child maltreatment on the diagnosis of depression, which is in line with these previous studies. Several studies examining impact of child maltreatment on disease outcomes saw attenuation of the relationship between child maltreatment and disease when adjusting for mental disorder (36). Our data supports existing evidence in indicating that child maltreatment is a risk factor for mental disorders which themselves may be mediators for disease severity of PAH (36).

QoL contributes to the evaluation of emerging PAH treatments as well as disease course. QoL in patients with PAH is severely impaired by clinical symptoms as well as emotional domains (20, 37). Mathai et al. (38) have found QoL to be a predictor of outcomes in PAH and thus a target for therapeutic interventions. Child maltreatment in patients with PAH correlate with worse QoL. This is in line previous studies such as Corso et al. who demonstrated significant and sustained losses of QoL in adulthood of persons who experienced child maltreatment (39, 40).

Several studies have demonstrated association between child maltreatment and cardiometabolic diseases such as systemic hypertension, ischemic heart disease, and chronic heart disease (41, 42). Many authors examined child maltreatment as a composite measure of adversity and reported a dose-response relationship between number of childhood adversities and heightened risk for cardiometabolic diseases. Overall, CTQ scores were associated with disease severity measured by WHO FC. In a subscore analysis of our study, we found that patients with higher scores in emotional neglect had a significantly higher chance of being in a higher WHO FC per CTQ point increase. While this impact might suggest negative influence on disease course, morbidity, and mortality model accuracy is comparatively low. Clinical signs of right heart failure, 6MWD, NT-proBNP/BNP, and WHO FC are strong indicators of disease severity and risk status in patients with PAH (23). Arguably these somatic factors play a more prominent role on disease severity with a minor role of child maltreatment and by association mental disorders.

### Limitations

Our paper has strength and limitations. Data for childhood maltreatment, mental health and QoL were derived using a self-administered, paper-based questionnaire. Reliable measurements of prevalence and severity of child maltreatment have yet relied on self-reports, showing discrepancies of rates compared to reports of child-protection agencies (43, 44). Median global prevalence rates differ substantially by maltreatment type, gender and by continent with varying number of studies and available data (45). Only two German PH-centers were involved in this study which might cause selection bias. This study was carried out between September 2019 and March 2020. In January, the Corona virus pandemic hit Germany and this study didn't control for any possible impact on mental disease during this time. Of note, Park et al. (46) have studied effects on first waves of corona virus pandemic on a subsample of this cohort and found minor changes in mental health due to the corona virus pandemic. However, a strength is relatively large sample size of PAH patients given that PAH is a rare disease as both participating centers are large university-based tertiary-level referral centers.

## Conclusion

In the present study, child maltreatment, in particular, subscores for emotional abuse and physical abuse was more prevalent in patients with PAH than in the general population. The presence of child maltreatment was associated with a higher likelihood of anxiety and depression and reduction in QoL. Child maltreatment in patients with PAH might play a role in disease severity. With wide ranges of long-term consequences of child maltreatment, screening for a history of child maltreatment can help physicians in assessment of patients' needs and treatment options. This should be taken into consideration in addition to more widely acknowledged impact of mental disorders and QoL on PAH. Psychosocial interventions as a treatment approach have been proven effective in patients with a history of child maltreatment (47–49). Integrating past experiences and psychological problems in the care and treatment of patients with PAH is recommended. The lasting consequences of child maltreatment warrant further investigation.

## Data Availability Statement

The raw data supporting the conclusions of this article will be made available by the authors, without undue reservation.

## Ethics Statement

The studies involving human participants were reviewed and approved by Hannover Medical School and University of Giessen and Marburg. The patients/participants provided their written informed consent to participate in this study.

## Author Contributions

DHP was responsible for study design, implementation of the study, data collection, data interpretation, and drafting the manuscript. TM was responsible for data collection and revising the manuscript. KK was responsible for study design, data interpretation, and revising the manuscript. JK was responsible for data interpretation and revising the manuscript. MR, HG, and HAG were responsible for implementation of the study and critically revising the manuscript. MH was responsible for implementation of the study, data interpretation, and critically revising the manuscript. KO was responsible for study design, implementation of the study, data interpretation, and critically revising the manuscript. JF was responsible for study design, implementation of the study, data collection, statistical analysis, data interpretation, and drafting the manuscript. All authors contributed to the article and approved the submitted version.

## Conflict of Interest

DHP has received honoraria for lectures and/or consultations from Janssen. KK has received honoraria for consultations and/or lectures from Eli Lilly, Janssen, Lundbeck, Neuraxpharm, Otsuka, Pfizer, Servier, Schwabe, Takeda, and Trommsdorff/Ferrer, Alexion, and CannaXan (advisory board). HG has received personal fees from Actelion, personal fees from AstraZeneca, personal fees from Bayer, personal fees from BMS, personal fees from GSK, personal fees from Janssen-Cilag, personal fees from Lilly, personal fees from MSD, personal fees from Novartis, personal fees from OMT, personal fees from Pfizer, personal fees from United Therapeutics, outside the submitted work. HG has received fees from Actelion, Bayer, Gilead, GSK, MSD, Pfizer, and United Therapeutics, outside the present work. HAG has received honoraria for lectures and/or consultations from Acceleron, Actelion, Bayer, GSK, Janssen, MSD and Pfizer, all outside the present study. KO has received honoraria for lectures and/or consultations from Acceleron, Actelion, Bayer, GSK, Janssen, MSD, United Therapeutics and Pfizer, all outside the present study. The remaining authors declare that the research was conducted in the absence of any commercial or financial relationships that could be construed as a potential conflict of interest.

## Publisher's Note

All claims expressed in this article are solely those of the authors and do not necessarily represent those of their affiliated organizations, or those of the publisher, the editors and the reviewers. Any product that may be evaluated in this article, or claim that may be made by its manufacturer, is not guaranteed or endorsed by the publisher.

## Abbreviations

6MWD: 6-minute walk distance
BNP: brain natriuretic peptide
CTQ: child trauma questionnaire
FC: World Health Organization functional class
HADS: Hospital Anxiety and Depression Scale
NT-proBNP: N-terminal fragment of probrain natriuretic peptide
NYHA: New York Heart Association
PAH: Pulmonary arterial hypertension
PEPPAH: Psychische Einschränkungen bei Patienten mit pulmonal arterieller Hypertonie (Prevalence of mental disorders and impact on quality of life in patients with pulmonary arteriaI hypertension)
QoL: quality of life
SD: Standard Deviation.

## References

[1] Galie N Humbert M Vachiery JL Gibbs S Lang I Torbicki A. 2015 ESC/ERS Guidelines for the diagnosis and treatment of pulmonary hypertension: The Joint Task Force for the Diagnosis and Treatment of Pulmonary Hypertension of the European Society of Cardiology (ESC) and the European Respiratory Society (ERS): Endorsed by: Association for European Paediatric and Congenital Cardiology (AEPC), International Society for Heart and Lung Transplantation (ISHLT). Eur Respir J. (2015) 46:903–75. 10.1183/13993003.01032-2015 26318161

[2] Hoeper MM Humbert M Souza R Idrees M Kawut SM Sliwa-Hahnle K. A global view of pulmonary hypertension. Lancet Respir Med. (2016) 4:306–22. 10.1016/S2213-2600(15)00543-3 26975810

[3] Shafazand S Goldstein MK Doyle RL Hlatky MA Gould MK. Health-related quality of life in patients with pulmonary arterial hypertension. Chest. (2004) 126:1452–9. 10.1378/chest.126.5.1452 15539712

[4] Fuge J Park DH von Lengerke T Richter MJ Gall H Ghofrani A. Impact of pulmonary arterial hypertension on employment, work productivity and quality of life - results of a cross-sectional multi-center study. Front Psychiatry. (2021). 10.3389/fpsyt.2021.781532 PMC8861193 35211036

[5] Sommer N Ghofrani HA Pak O Bonnet S Provencher S Sitbon O. Current and future treatments of pulmonary arterial hypertension. Br J Pharmacol. (2021) 178:6–30. 10.1111/bph.15016 32034759

[6] Armstrong I Billings C Kiely DG Yorke J Harries C Clayton S. The patient experience of pulmonary hypertension: a large cross-sectional study of UK patients. BMC Pulm Med. (2019) 19:67. 10.1186/s12890-019-0827-5 30898139 PMC6429756

[7] Zhai Z Zhou X Zhang S Xie W Wan J Kuang T. The impact and financial burden of pulmonary arterial hypertension on patients and caregivers: results from a national survey. Medicine (Baltimore). (2017) 96:e6783. 10.1097/MD.0000000000006783 28953608 PMC5626251

[8] Guillevin L Armstrong I Aldrighetti R Howard LS Ryftenius H Fischer A. Understanding the impact of pulmonary arterial hypertension on patients' and carers' lives. Eur Respir Rev. (2013) 22:535–42. 10.1183/09059180.00005713 24293469 PMC9639179

[9] Von Visger TT Kuntz KK Phillips GS Yildiz VO Sood N. Quality of life and psychological symptoms in patients with pulmonary hypertension. Heart Lung. (2018) 47:115–21. 10.1016/j.hrtlng.2017.12.004 29361341

[10] Larisch A Neeb C de Zwaan M Pabst C Tiede H Ghofrani A. [Mental distress and wish for psychosomatic treatment of patients with pulmonary hypertension]. Psychother Psychosom Med Psychol. (2014) 64:384–9. 10.1055/s-0034-1377013 25029250

[11] Jacobi F Hofler M Siegert J Mack S Gerschler A Scholl L. Twelve-month prevalence, comorbidity and correlates of mental disorders in Germany: the Mental Health Module of the German Health Interview and Examination Survey for Adults (DEGS1-MH). Int J Methods Psychiatr Res. (2014) 23:304–19. 10.1002/mpr.1439 24729411 PMC6878234

[12] Olsson KM Meltendorf T Fuge J Kamp JC Park DH Richter MJ. Prevalence of mental disorders and impact on quality of life in patients with pulmonary arterial hypertension. Front Psychiatry. (2021) 12:667602. 10.3389/fpsyt.2021.667602 34135787 PMC8200462

[13] Nelson J Klumparendt A Doebler P Ehring T. Childhood maltreatment and characteristics of adult depression: meta-analysis. Br J Psychiatry. (2017) 210:96–104. 10.1192/bjp.bp.115.180752 27908895

[14] Leeb R Paulozzi L Melanson C Simon T Arias I. Child Maltreatment Surveillance. Uniform Definitions for Public Health and Recommended Data Elements. Atlanta: Centers for Disease Control and Prevention (2008).

[15] Walker EA Gelfand A Katon WJ Koss MP Von Korff M Bernstein D. Adult health status of women with histories of childhood abuse and neglect. Am J Med. (1999) 107:332–9. 10.1016/S0002-9343(99)00235-1 10527034

[16] Clemens V Huber-Lang M Plener PL Brahler E Brown RC Fegert JM. Association of child maltreatment subtypes and long-term physical health in a German representative sample. Eur J Psychotraumatol. (2018) 9:1510278. 10.1080/20008198.2018.1510278 30220980 PMC6136347

[17] Ho FK Celis-Morales C Gray SR Petermann-Rocha F Lyall D Mackay D. Child maltreatment and cardiovascular disease: quantifying mediation pathways using UK Biobank. BMC Med. (2020) 18:143. 10.1186/s12916-020-01603-z 32527275 PMC7291652

[18] Shields ME Hovdestad WE Gilbert CP Tonmyr LE. Childhood maltreatment as a risk factor for COPD: findings from a population-based survey of Canadian adults. Int J Chron Obstruct Pulmon Dis. (2016) 11:2641–50. 10.2147/COPD.S107549 27822027 PMC5087754

[19] Proskynitopoulos PJ Heitland I Glahn A Bauersachs J Westhoff-Bleck M Kahl KG. Prevalence of child maltreatment in adults with congenital heart disease and its relationship with psychological well-being, health behavior, and current cardiac function. Front Psychiatry. (2021) 12:686169. 10.3389/fpsyt.2021.686169 34381388 PMC8350035

[20] McGoon MD Ferrari P Armstrong I Denis M Howard LS Lowe G. The importance of patient perspectives in pulmonary hypertension. Eur Respir J. (2019) 53:1801919. 10.1183/13993003.01919-2018 30545977 PMC6351339

[21] Kylhammar D Kjellstrom B Hjalmarsson C Jansson K Nisell M Soderberg S. A comprehensive risk stratification at early follow-up determines prognosis in pulmonary arterial hypertension. Eur Heart J. (2018) 39:4175–81. 10.1093/eurheartj/ehx257 28575277

[22] Benza RL Gomberg-Maitland M Miller DP Frost A Frantz RP Foreman AJ. The REVEAL Registry risk score calculator in patients newly diagnosed with pulmonary arterial hypertension. Chest. (2012) 141:354–62. 10.1378/chest.11-0676 21680644

[23] Hoeper MM Kramer T Pan Z Eichstaedt CA Spiesshoefer J Benjamin N. Mortality in pulmonary arterial hypertension: prediction by the 2015 European pulmonary hypertension guidelines risk stratification model. Eur Respir J. (2017) 50:1700740. 10.1183/13993003.00740-2017 28775047

[24] Wingenfeld K Spitzer C Mensebach C Grabe HJ Hill A Gast U. [The German version of the Childhood Trauma Questionnaire (CTQ): preliminary psychometric properties]. Psychother Psychosom Med Psychol. (2010) 60:442–50. 10.1055/s-0030-1247564 20200804

[25] Iffland B Brahler E Neuner F Hauser W Glaesmer H. Frequency of child maltreatment in a representative sample of the German population. BMC Public Health. (2013) 13:980. 10.1186/1471-2458-13-980 24139055 PMC3854006

[26] Zigmond AS Snaith RP. The hospital anxiety and depression scale. Acta Psychiatr Scand. (1983) 67:361–70. 10.1111/j.1600-0447.1983.tb09716.x 6880820

[27] Bjelland I Dahl AA Haug TT Neckelmann D. The validity of the Hospital Anxiety and Depression Scale. J Psychosom Res. (2002) 52:69–77. 10.1016/S0022-3999(01)00296-3 11832252

[28] Lowe B. Comparative validity of three screening questionnaires for DSM-IV depressive disorders and physicians' diagnoses. J Affect Disord. (2004) 78:131–40. 10.1016/S0165-0327(02)00237-9 14706723

[29] Harper A Power M. Development of the World Health Organization WHOQOL-BREF quality of life assessment. The WHOQOL Group. Psychol Med. (1998) 28:551–8. 10.1017/S0033291798006667 9626712

[30] Rosen AL Handley ED Cicchetti D Rogosch FA. The impact of patterns of trauma exposure among low income children with and without histories of child maltreatment. Child Abuse Negl. (2018) 80:301–11. 10.1016/j.chiabu.2018.04.005 29674290 PMC5953839

[31] Norman RE Byambaa M De R Butchart A Scott J Vos T. The long-term health consequences of child physical abuse, emotional abuse, and neglect: a systematic review and meta-analysis. PLoS Med. (2012) 9:e1001349. 10.1371/journal.pmed.1001349 23209385 PMC3507962

[32] Etain B Mathieu F Henry C Raust A Roy I Germain A. Preferential association between childhood emotional abuse and bipolar disorder. J Trauma Stress. (2010) 23:376–83. 10.1002/jts.20532 20564371

[33] Huh HJ Kim SY Yu JJ Chae JH. Childhood trauma and adult interpersonal relationship problems in patients with depression and anxiety disorders. Ann Gen Psychiatry. (2014) 13:26. 10.1186/s12991-014-0026-y 25648979 PMC4304140

[34] Cohen P Brown J Smailes E. Child abuse and neglect and the development of mental disorders in the general population. Dev Psychopathol. (2002) 13:981–99. 10.1017/S0954579401004126 11771917

[35] Gamble SA Talbot NL Duberstein PR Conner KR Franus N Beckman AM. Childhood sexual abuse and depressive symptom severity: the role of neuroticism. J Nerv Ment Dis. (2006) 194:382–5. 10.1097/01.nmd.0000218058.96252.ac 16699389

[36] Basu A McLaughlin KA Misra S Koenen KC. Childhood maltreatment and health impact: the examples of cardiovascular disease and type 2 diabetes mellitus in adults. Clin Psychol (New York). (2017) 24:125–39. 10.1111/cpsp.12191 28867878 PMC5578408

[37] Taichman DB Shin J Hud L Archer-Chicko C Kaplan S Sager JS. Health-related quality of life in patients with pulmonary arterial hypertension. Respir Res. (2005) 6:92. 10.1186/1465-9921-6-92 16092961 PMC1208953

[38] Mathai SC Suber T Khair RM Kolb TM Damico RL Hassoun PM. Health-related quality of life and survival in pulmonary arterial hypertension. Ann Am Thorac Soc. (2016) 13:31–9. 10.1513/AnnalsATS.201412-572OC 26492065 PMC4722843

[39] Corso PS Edwards VJ Fang X Mercy JA. Health-related quality of life among adults who experienced maltreatment during childhood. Am J Public Health. (2008) 98:1094–100. 10.2105/AJPH.2007.119826 18445797 PMC2377283

[40] Jud A Landolt MA Tatalias A Lach LM Lips U. Health-related quality of life in the aftermath of child maltreatment: follow-up study of a hospital sample. Qual Life Res. (2013) 22:1361–9. 10.1007/s11136-012-0262-z 22996648

[41] Suglia SF Clark CJ Boynton-Jarrett R Kressin NR Koenen KC. Child maltreatment and hypertension in young adulthood. BMC Public Health. (2014) 14:1149. 10.1186/1471-2458-14-1149 25374338 PMC4240900

[42] Schafer MH Ferraro KF. Childhood misfortune as a threat to successful aging: avoiding disease. Gerontologist. (2012) 52:111–20. 10.1093/geront/gnr071 21746836 PMC3265554

[43] Fergusson D Horwood L Woodward L. The stability of child abuse reports: a longitudinal study of the reporting behaviour of young adults. Psychol Med. (2000) 30:529–44. 10.1017/S0033291799002111 10883709

[44] MacMillan HL Jamieson E Walsh CA. Reported contact with child protection services among those reporting child physical and sexual abuse: results from a community survey. Child Abuse Negl. (2003) 27:1397–408. 10.1016/j.chiabu.2003.06.003 14644057

[45] Moody G Cannings-John R Hood K Kemp A Robling M. Establishing the international prevalence of self-reported child maltreatment: a systematic review by maltreatment type and gender. BMC Public Health. (2018) 18:1164. 10.1186/s12889-018-6044-y 30305071 PMC6180456

[46] Park DH Fuge J Meltendorf T Kahl KG Richter MJ Gall H. Impact of SARS-CoV-2-pandemic on mental disorders and quality of life in patients with pulmonary arterial hypertension. Front Psychiatry. (2021) 12:668647. 10.3389/fpsyt.2021.668647 34248706 PMC8263927

[47] Cohen JA Mannarino AP Murray LK Igelman R. Psychosocial interventions for maltreated and violence-exposed children. J Soc Issues. (2006) 62:737–66. 10.1111/j.1540-4560.2006.00485.x

[48] Domhardt M Baumeister H. Psychotherapy of adjustment disorders: current state and future directions. World J Biol Psychiatry. (2018) 19:S21–S35. 10.1080/15622975.2018.1467041 30204563

[49] Goodnight JRM Ragsdale KA Rauch SAM Rothbaum BO. Psychotherapy for PTSD: an evidence-based guide to a theranostic approach to treatment. Prog Neuropsychopharmacol Biol Psychiatry. (2019) 88:418–26. 10.1016/j.pnpbp.2018.05.006 29786514

